# N_2_O Reduction by *Gemmatimonas aurantiaca* and Potential Involvement of *Gemmatimonadetes* Bacteria in N_2_O Reduction in Agricultural Soils

**DOI:** 10.1264/jsme2.ME21090

**Published:** 2022-04-12

**Authors:** Mamoru Oshiki, Yuka Toyama, Toshikazu Suenaga, Akihiko Terada, Yasuhiro Kasahara, Takashi Yamaguchi, Nobuo Araki

**Affiliations:** 1 Department of Civil Engineering, National Institute of Technology, Nagaoka College, Nagaoka, Niigata, Japan; 2 Division of Environmental Engineering, Faculty of Engineering, Hokkaido University, Sapporo, Hokkaido, Japan; 3 Department of Chemical Engineering, Hiroshima University, Higashi-hiroshima, Hiroshima, Japan; 4 Department of Applied Physics and Chemical Engineering, Tokyo University of Agriculture and Technology, Koganei, Tokyo, Japan; 5 Institute of Low Temperature Science, Hokkaido University, Sapporo, Hokkaido, Japan; 6 Department of Science of Technology Innovation, Nagaoka University of Technology, Nagaoka, Niigata, Japan

**Keywords:** Nitrous oxide (N_2_O) reduction, nitrous oxide reductase *nosZ*, agricultural soil, *Gemmatimonadetes*, *Gemmatimonas aurantiaca*

## Abstract

Agricultural soil is the primary N_2_O sink limiting the emission of N_2_O gas into the atmosphere. Although *Gemmatimonadetes* bacteria are abundant in agricultural soils, limited information is currently available on N_2_O reduction by *Gemmatimonadetes* bacteria. Therefore, the effects of pH and temperature on N_2_O reduction activities and affinity constants for N_2_O reduction were examined by performing batch experiments using an isolate of *Gemmatimonadetes* bacteria, *Gemmatimonas aurantiaca* (NBRC100505^T^). *G. aurantiaca* reduced N_2_O at pH 5–9 and 4–50°C, with the highest activity being observed at pH 7 and 30°C. The affinity constant of *G. aurantiaca* cells for N_2_O was 4.4‍ ‍μM. The abundance and diversity of the *Gemmatimonadetes* 16S rRNA gene and *nosZ* encoding nitrous oxide reductase in agricultural soil samples were also investigated by quantitative PCR (qPCR) and amplicon sequencing ana­lyses. Four N_2_O-reducing agricultural soil samples were assessed, and the copy numbers of the *Gemmatimonadetes* 16S rRNA gene (clades G1 and G3), *nosZ* DNA, and *nosZ* mRNA were 8.62–9.65×10^8^, 5.35–7.15×10^8^, and 2.23–4.31×10^9^ copies (g dry soil)^–1^, respectively. The abundance of the *nosZ* mRNA of *Gemmatimonadetes* bacteria and OTU91, OUT332, and OTU122 correlated with the N_2_O reduction rates of the soil samples tested, suggesting N_2_O reduction by *Gemmatimonadetes* bacteria. *Gemmatimonadetes* 16S rRNA gene reads affiliated with OTU4572 and OTU3759 were predominant among the soil samples examined, and these *Gemmatimonadetes* OTUs have been identified in various types of soil samples.

N_2_O gas is a notorious greenhouse gas because of its strong global warming potential (265-fold greater than that of carbon dioxide) and persistence in the atmosphere (*ca*. 114 years) (Ravishankara *et al.*, 2009; Montzka *et al.*, 2011; IPCC, 2014). N_2_O gas also contributes to the loss of stratospheric ozone and has been recognized as the dominant ozone-depleting substance (Ravishankara *et al.*, 2009; Montzka *et al.*, 2011). Terrestrial soils greatly contribute to N_2_O emissions (*i.e.*, 6–7 ton g year^–1^, corresponding to *ca.* 60% of total N_2_O emissions) (Butterbach-Bahl *et al.*, 2013; Cui *et al.*, 2013; Tian *et al.*, 2020), and N_2_O gas is discharged from soils as a net result of soil N_2_O production and consumption (Holtan-Hartwig *et al.*, 2000). The application of nitrogenous fertilizers to agricultural soils is a common practice, but markedly increases N_2_O emissions from agricultural soils (Seitzinger *et al.*, 2000; Liu and Greaver, 2009; Bouwman *et al.*, 2013). More than 50% of the annual consumption of nitrogenous fertilizers is currently derived from urea-based fertilizer consumption (Glibert *et al.*, 2014), and urea is hydrolyzed to ammonia and carbon dioxide by ureolytic microorganisms (Oshiki *et al.*, 2018). The ammonia produced is subsequently oxidized to nitrite and/or nitrate by nitrification, followed by the reduction of nitrite and nitrate to nitrogen gas by denitrification and/or anammox processes (Isobe and Ohte, 2014; Oshiki *et al.*, 2016). N_2_O gas is produced biotically and abiotically by these nitrification and denitrification processes (Ishii *et al.*, 2011). Regarding the consumption of N_2_O, the biological reduction of N_2_O is the only reaction that acts as a N_2_O sink. This N_2_O reduction reaction is catalyzed by the multicopper enzyme, nitrous oxide reductase (NosZ), which catalyzes the reduction of two electrons of N_2_O to produce N_2_ (Richardson *et al.*, 2009; Simon and Klotz, 2013). The abundance and diversity of *nosZ* in soils have received a great deal of attention as a functional gene marker of N_2_O reducers. *nosZ* has been found in various bacterial and archaeal genomes and classified into two phylogenetically distinct clades, *nosZ* clades I and II, based on sequence homology (Sanford *et al.*, 2012; Jones *et al.*, 2013). The abundance and diversity of *nosZ* clade I have been investigated in various types of soils, whereas those of *nosZ* clade II were largely overlooked until 2012 (Sanford *et al.*, 2012). *nosZ* clade II was overlooked because the previously published oligonucleotide primer set (*i.e.*, the nosZ1F and nosZ1R primers) (Henry *et al.*, 2006) utilized for the PCR amplification of environmental *nosZ* sequences did not cover the sequence divergence of *nosZ* clade II. Recent molecular ana­lyses, including quantitative PCR (qPCR), an amplicon sequencing ana­lysis of *nosZ* clade II, and a metagenomic ana­lysis of soil DNA, revealed the abundant distribution of *nosZ* clade II in various types of soils, similar to *nosZ* clade I (Sanford *et al.*, 2012; Jones *et al.*, 2013; Jones *et al.*, 2014; Orellana *et al.*, 2014; Domeignoz-Horta *et al.*, 2015; Samad *et al.*, 2016; Juhanson *et al.*, 2017). In addition to their phylogenetic differences, N_2_O reducers carrying *nosZ* clade II showed an affinity for N_2_O that was up to two orders of magnitude higher than those carrying *nosZ* clade I (Yoon *et al.*, 2016; Suenaga *et al.*, 2018), suggesting their significant contribution to N_2_O mitigation from soils because the concentration of N_2_O is generally low in soils (*i.e.*, typically less than 1‍ ‍μM) (Schreiber *et al.*, 2012). A linear regression ana­lysis (Domeignoz-Horta *et al.*, 2015; 2018; Samad *et al.*, 2016) and structural equation modeling and network ana­lysis (Jones *et al.*, 2014) of the gene abundance and diversity of *nosZ* clade II in terrestrial soils indicated the greater contribution of N_2_O reducers carrying *nosZ* clade II to the soil N_2_O sink capacity than those carrying *nosZ* clade I.

The phylum *Gemmatimonadetes* is currently recognized as one of the nine dominant soil phyla (Janssen, 2006) because the *Gemmatimonadetes* 16S rRNA gene has been frequently and abundantly detected in various terrestrial environments (Janssen, 2006; DeBruyn *et al.*, 2011). The phylum *Gemmatimonadetes* contains phylogenetically diverse bacterial members that have been classified into at least five sublineages (Hanada and Sekiguchi, 2014), and the following isolates have been described: *Gemmatimonas aurantiaca* (Zhang *et al.*, 2003), *G. phototrophica* (Zeng *et al.*, 2017), *G. groenlandica* (Zeng *et al.*, 2021), *Gemmatirosa kalamazoonensis* (DeBruyn *et al.*, 2013), *Roseisolibacter agri* (Pascual *et al.*, 2018), and *Longimicrobium terrae* (Pascual *et al.*, 2016). *G. aurantiaca* and *Gt. kalamazoonesis* carry *nosZ* clade II (accession numbers AP009153.1 and CP007128.1, respectively), and N_2_O reduction by *G. aurantiaca* was recently demonstrated in a culture-dependent manner (Park *et al.*, 2017; Chee-Sanford *et al.*, 2019). Although *G. aurantiaca* has been characterized as an obligate aerobic heterotroph (Zhang *et al.*, 2003), this bacterium reduced N_2_O under not only aerobic, but also microaerobic and anoxic conditions when partially oxic conditions were present (Chee-Sanford *et al.*, 2019). *G. aurantiaca* cells transcribed *nosZ* when they reduced N_2_O (Park *et al.*, 2017), suggesting that the abundance of *Gemmatimonadetes nosZ* mRNA correlates with that of a metabolically-active *Gemmatimonadetes* bacterial population. The metabolic capability of N_2_O reduction by *G. aurantiaca* and the widespread distribution of *Gemmatimonadetes* bacteria in various types of soils including agricultural soils (Jones *et al.*, 2014; Orellana *et al.*, 2014; Samad *et al.*, 2016) led us to expect the contribution of *Gemmatimonadetes* bacteria to N_2_O mitigation from soils. However, the physiological characteristics of *G. aurantiaca* for N_2_O reduction have not yet been examined in detail, and limited information is currently available on the involvement of *Gemmatimonadetes* bacteria in N_2_O reduction in soils. Physiological pH and temperature ranges and affinity constants are key physiological information for obtaining a more detailed understanding of microbial activities in natural and man-made ecosystems (Oshiki *et al.*, 2016).

Therefore, the present study investigated 1) the physiological characteristics of *G. aurantiaca* associated with N_2_O reduction, and 2) the involvement of *Gemmatimonadetes* bacteria in N_2_O reduction in agricultural soils. The effects of pH and temperature on N_2_O reduction activities and affinity constants for N_2_O reduction by *G. aurantiaca* were examined by performing batch incubations and assessing N_2_O reduction activities. The involvement of *Gemmatimonadetes* bacteria in N_2_O reduction in agricultural soils was then analyzed by a soil incubation experiment in which the relationship between the N_2_O reduction rates of soils and the abundance of *Gemmatimonadetes* bacteria was investigated. Agricultural soil samples were incubated with the addition of ^15^NO_3_^–^ to evaluate N_2_O reduction rates, and the abundance and diversity of the *Gemmatimonadetes* 16S rRNA gene and *nosZ* in the soil samples tested were evaluated by qPCR and amplicon sequencing ana­lyses. The above DNA-based ana­lyses potentially detect metabolically-inactive *Gemmatimonadetes* bacteria; therefore, qPCR and amplicon sequencing ana­lyses of *Gemmatimonadetes nosZ* mRNA were also conducted, and the relationship between N_2_O reduction rates and the abundance of *nosZ* mRNA was investigated. This is the first study to show a correlation between N_2_O reduction rates and the abundance of *Gemmatimonadetes nosZ* mRNA in agricultural soils, and the potential involvement of *Gemmatimonadetes* bacteria in N_2_O reduction in soil is discussed.

## Materials and Methods

### Bacterial culture and incubation conditions

*G. aurantiaca* (NBRC100505^T^) cells were cultivated aerobically at 30°C with shaking at 90‍ ‍rpm. NBRC822 medium (L^–1^: glucose, 0.5 g; peptone [BD Difco, Becton Dickinson and company], 0.5‍ ‍g; yeast extract [BD Difco], 0.5 g; sodium glutamate, 0.5 g; KH_2_PO_4_, 0.44 g; [NH_4_]_2_SO_4_, 0.1 g; MgSO_4_·7H_2_O, 0.1 g; pH 7.0) was used for cultivation. Stationary-phase cells were harvested by centrifugation (13,420×*g*, 10‍ ‍min), washed, and resuspended in fresh NBRC822 medium. The cell suspension was subjected to the following activity tests.

### Effects of pH and temperature on the N_2_O reduction activity of G. aurantiaca

*G. aurantiaca* cells were incubated at pH 5 to 10 and at 4 to 80°C, and the consumption of N_2_O was examined. Three milliliters of NBRC822 medium was dispensed into 7.7-mL serum glass vials (Nichiden-Rika Glass), which were then sealed with butyl rubber stoppers and aluminum caps. The pH of NBRC822 medium was adjusted in the range of pH 5 to 10 by adding the following pH buffer at a final concentration of 20‍ ‍mM: 2-morpholinoethanesulfonic acid, monohydrate (MES) for pH 5 to 6.5, 2-[4-(2-hydroxyethyl)-1-piperazinyl]ethanesulfonic acid (HEPES) for pH 7 to 7.5, and N-[Tris(hydroxymethyl)methyl]glycine (Tricine) for pH 8 to 10. After purging the liquid phase with argon gas for 3‍ ‍min, the headspace was replaced with pure He gas (>99.99995%). One hundred microliters of the *G. aurantiaca* cell suspension and N_2_O gas (GL Science) were injected into the vials at a final concentration of 0.1–0.5‍ ‍mg protein mL^–1^ and 180‍ ‍nmol (N vial)^–1^, respectively, using a gas tight syringe. The vials were incubated at 4 to 80°C in the dark, and changes in N_2_O concentrations in the headspace over time were examined.

Partial oxic conditions were essential for initiating N_2_O reduction by *G. aurantiaca*, which occurred after the depletion of O_2_ (Park *et al.*, 2017). Although O_2_ was not added externally to the above vials, certain amounts of oxygen were available in the vials due to 1) the incomplete removal of O_2_ (*e.g.*, 3‍ ‍min of an argon gas purge) and 2) the carryover of O_2_ from the inoculum, which were adequate to initiate N_2_O reduction by *G. aurantiaca*.

### Affinity of G. aurantiaca cells for N_2_O

The value of *K_s_* for N_2_O was calculated based on the N_2_O consumption rate assessed using a N_2_O-specific microsensor (N_2_O-MR) and micro-respirometric system (Unisense) as previously described (Suenaga *et al.*, 2018). Briefly, *G. aurantiaca* cells suspended in NBRC822 medium (pH 7) were dispensed into a 3.0-mL closed chamber (Unisense) and stirred with a dedicated stirrer bar at 300‍ ‍rpm. N_2_O-saturated NBRC 822 medium (27–24‍ ‍mM N_2_O at 20–25°C) was added using a Hamilton syringe to reach a final N_2_O concentration of 30‍ ‍μM. The chamber was incubated at 30°C, and the N_2_O concentration in the liquid phase was continuously monitored using SensorTrace Suite ver.2.8.0 (Unisense). N_2_O concentration profiles were smoothed in Sigma Plot 13.0 to remove high frequency noise. The value of *K_s_* was assessed by fitting N_2_O concentrations and instantaneous consumption rates to the Michaelis-Menten equation using the solver function in Microsoft Excel. N_2_O-MR was calibrated prior to incubations using aqueous N_2_O solution as described in the manual provided by the supplier.

### N_2_O reduction by agricultural soils

Agricultural soils were incubated with the addition of ^15^NO_3_^–^, and the production of ^15-15^N_2_O and ^15-15^N_2_ gas was examined (Fukushi *et al.*, 1984; Shan *et al.*, 2016). Agricultural soils (designated Soils A, B, C, and D) were collected at four sites in Nagaoka city, Niigata, Japan (Table S1). Surface layers (0 to 5‍ ‍cm) were collected from five spots at each site (5×1 m) and sieved (pore diameter, 2‍ ‍mm) to remove concomitant gravel. The 10 g-wet of the sieved soils was dispensed into 50-mL serum glass vials, Na^15^NO_3_^–^ was added at a final concentration of 71.4‍ ‍μmol (N vial)^–1^, and the vials were sealed using a butyl rubber stopper and aluminum seal. The head space was replaced with pure He gas, and the vials were incubated in the dark at 30°C in quadruplicate. After an incubation for 69 h, incubated soils were collected from the vials using a sterile spatula and subjected to total DNA and RNA extraction.

### Total DNA and RNA extractions

Total DNA and RNA extractions were performed using a Power Soil DNA Isolation and RNA PowerSoil Total RNA Isolation Kit (Qiagen Japan), respectively, according to the manufacturer’s protocols. Extracted RNA was transcribed to cDNA using a random 6mer primer and Prime Script RT Reagent Kit (TaKaRa Bio) (Kobayashi *et al.*, 2017).

### qPCR assay

The copy numbers of the 1) prokaryotic and 2) *Gemmatimonadetes* 16S rRNA gene (clades G1 and G3), 3) *Gemmatimonadetes nosZ* DNA, and 4) *Gemmatimonadetes nosZ* mRNA (*i.e.*, synthesized cDNA) were assessed using the MiniOpticon Real-Time PCR System (Bio-Rad, Hercules). The reaction mixture (20‍ ‍μL) contained the KAPA SYBR FAST qPCR master mix (Nippon Genetics) (10‍ ‍μL), 0.8‍ ‍μL of each forward and reverse primer (10‍ ‍μM), and 1.6‍ ‍μL of extracted DNA or the synthesized cDNA template. The oligonucleotide primers used for PCR amplification were as follows: 515F (5′-GTGCCAGCMGCCGCGGTAA-3′) and 806r (5′-GGACTACHVGGGTWTCTAAT-3′) for the prokaryotic 16S rRNA gene (Caporaso *et al.*, 2011), G1G3-673F (5′-GAATGCGTAGAGATCC-3′) and 907r (5′-CCGTCAATTCMTTTRAGTTT-3′) for the *Gemmatimonadetes* 16S rRNA gene affiliated to clades G1 and G3, which were previously described by DeBruyn *et al.*, (2011), and nosZ-123-145-F (5′-AACAAGAACCSAAGGAYCG-3′) and nosZ-481-499-R (5′-ATRTCCCARTCCTGYTC-3′) for *Gemmatimonadetes nosZ* (the present study). Cycling conditions were as follows: 95°C for 30 s; 40 cycles at 95°C for 5‍ ‍s and 55°C for 10 s; and 65°C to 95°C in increments of 0.5°C for the melting curve ana­lysis. Negative controls (*i.e.*, distilled water and an RNA template that was not reverse transcribed) were subjected to qPCR in parallel, and no amplicon was obtained from these negative controls. The genomic DNA of *G. aurantiaca* with a single copy of the 16S rRNA gene and *nosZ* was used as a standard for quantification. DNA concentrations were measured using the Qubit dsDNA BR assay kit and Qubit 3.0 fluorospectrometer (Thermo Fisher Scientific). Genomic DNA was serially diluted with distilled water to concentrations of 10^5^ to 10^0^ copies μL^–1^.

The nosZ-123-145-F and nosZ-481-499-R primers were newly designed in the present study. We attempted to amplify the partial sequences of clade II *nosZ* with the nosZ-II-F and nosZ-II-R primers (Jones *et al.*, 2013). However, no specific amplicon was obtained by PCR amplification using nosZ-II-F and nosZ-II-R primers from the DNA and cDNA samples prepared from the soil samples tested, even after the optimization of PCR conditions (*i.e.*, Taq polymerase and the addition of DMSO and betaine) and cycling parameters (annealing temperature and extension time). A similar phenomenon was previously reported (Samad *et al.*, 2016); therefore, we designed a new set of oligonucleotide primers for the specific detection of *Gemmatimonadetes nosZ*. The nosZ-123-145-F and nosZ-481-499-R primers were designed by performing a blastn search using the *G. aurantiaca nosZ* sequence as a query sequence against the *nr* database (NCBI, accessed on December 2016). The top 500 *nosZ* sequence hits were aligned using MUSCLE software under default conditions (18 iterations) (Edgar, 2004), and the conserved regions suitable for PCR primer design were manually examined. The coverage of the designed primers was examined by aligning *Gemmatimonadetes nosZ* sequences and the designed *nosZ* primer sequences, and by counting the numbers of primer-template mismatches.

### Amplicon sequencing ana­lysis of the 16S rRNA gene and nosZ

The prokaryotic 16S rRNA gene and *Gemmatimonadetes nosZ* were amplified by PCR using the above oligonucleotide primers containing Illumina tag sequences at the 5′ end of the forward and reverse primers (5′-TCGTCGGCAGCGTCAGATGTGTATAAGAGACAG-3′ and 5′-GTCTCGTGGGCTCGGAGATGTGTATAAGAGACAG-3′, respectively). The PCR mixture had a volume of 20‍ ‍μL and contained 2‍ ‍μL of extracted DNA or synthesized cDNA, oligonucleotide primers (1‍ ‍μM each), dNTPs (200‍ ‍μM), 2% (v/v) DMSO, 1×PCR buffer, and ExTaq polymerase (0.025‍ ‍U‍ ‍μL^–1^). Thermal cycling conditions were as follows: 35 cycles at 98°C for 10‍ ‍s, followed by 55°C for 30‍ ‍s, then 72°C for 30 s; and 72°C for 10‍ ‍min. PCR products were purified using the FastGene Gel/PCR Extraction Kit (Nippon Genetics). Purified PCR products were tagged with a sample-unique index and Illumina adapter sequences at their 5' ends (Nextera XT Index Kit v2; Illumina) by PCR. The PCR reaction mixture (20‍ ‍μL) contained 1×KAPA HiFi HS ReadyMix (Nippon Genetics), 1‍ ‍μL of each forward and reverse primer (10‍ ‍μM), and 2‍ ‍μL of the recovered PCR products. PCR was run under the following cycling conditions: 95°C for 3‍ ‍min, 10 cycles of 95°C for 20‍ ‍s, 65°C for 15‍ ‍s, and 72°C for 1‍ ‍min; and 72°C for 5‍ ‍min. After agarose gel electrophoresis, PCR products were excised from the gel and purified using the Agencourt AMPure XP Kit (Beckman Coulter). The tagged amplicons were pooled and sequenced on an Illumina MiSeq platform in a 250-bp paired-end sequencing reaction using the v2 reagent kit (Illumina).

### Bioinformatics

The generated 16S rRNA gene and *nos*Z sequence reads were processed for the removal of adapter sequences using cutadapt and for quality trimming using Trimmomatic v0.33 (Bolger *et al.*, 2014). Reads containing <50 bp or those associated with an average Phred-like quality score <30 were removed. Paired-end sequence reads were assembled in the paired-end assembler of the Illumina sequence software package (PANDAseq) (Masella *et al.*, 2012). The *nos*Z reads obtained were subjected to a blastn search (threshold *e*-value; 10^–10^) against the 22,647 *nosZ* sequences downloaded from the fungene database (http://fungene.cme.msu.edu/) to remove non-*nosZ* sequences. Regarding 16S rRNA, assembled sequence reads with ≥97% sequence identity were grouped into OTUs by UCLUST (Edgar, 2004). The phylogenetic affiliations of the OTUs were identified by a blastn search against reference sequences in Greengenes database version 13_5 (DeSantis *et al.*, 2006) and in the *nr* database (NCBI). Regarding *nosZ*, sequence reads with ≥80% sequence identity were grouped into OTUs as previously reported (Palmer *et al.*, 2009), and their phylogenetic affiliation was examined by a blastn search against the nr database. Putative chimeric sequences were removed using UCHIME (Edgar *et al.*, 2011). Alpha diversity indices (observed species, Chao1, Good’s coverage, and Simpson’s index) were calculated in QIIME (Caporaso *et al.*, 2010). Chao1 was computed at sampling depths of 6,500 and 2,700 reads for 16S rRNA and *nosZ*, respectively. The alignment of nucleic acid and protein sequences was performed using MUSCLE software (Edgar, 2004) with 18 iterations, and a phylogenetic tree was constructed in MEGA 7.0.26 (Kumar *et al.*, 2016) using the maximum likelihood method (Jones-Taylor-Thornton model).

### Chemical ana­lysis

^14-14^N_2_O, ^15-15^N_2_O, and ^15-15^N_2_ concentrations were measured by gas chromatography-mass spectrometry (GS/MS) as previously described (Isobe *et al.*, 2011; Yoshinaga *et al.*, 2011). Ten microliters of the headspace gas was collected using a 100-μL gas-tight glass syringe and immediately injected into the gas chromatograph GCMS-QP 2010 SE (Shimadzu) equipped with a fused silica capillary column (Agilent Technologies). Peaks at *m/z*=30, 44, and 46, corresponding to ^15-15^N_2_, ^14-14^N_2_O, and ^15-15^N_2_O, were monitored, and concentrations were calculated using standard curves prepared using the standard ^14-14^N_2_O gas for both ^14-14^N_2_O and ^15-15^N_2_O (Shimakyu) and the ^15-15^N_2_ gas (Cambridge Isotope Laboratories).

Biomass concentrations were measured by the Lowry method using the DC-protein assay kit (Bio-Rad) as previously reported (Oshiki *et al.*, 2011). Bovine serum albumin was used to prepare calibration curves.

### Correlation ana­lysis between N_2_O reduction rates and physicochemical parameters or the abundance of the Gemmatimonadetes 16S rRNA gene or nosZ

A linear regression ana­lysis was performed using Microsoft Excel 16.57 to assess the coefficient of determination (*R^2^*) between N_2_O reduction rates (16.6, 5.0, 24.3, and 9.5‍ ‍nmol-N [g dry soil]^–1^ h^–1^ in Soils A, B, C, and D, respectively, see below) and physicochemical parameters (Table S1) or the copy numbers of the prokaryotic 16S rRNA gene, *Gemmatimonadetes* 16S rRNA gene, *Gemmatimonadetes nosZ* DNA, and *Gemmatimonadetes nosZ* mRNA (copies [g dry soil]^–1^) (Fig. 4). The copy numbers of *Gemmatimonadetes nosZ* mRNA affiliated to specific OTUs were calculated by multiplying the relative abundance of each OTU (%) by the copy numbers of *Gemmatimonadetes nosZ* mRNA.

### Nucleotide sequence accession number

Raw sequence data obtained in the amplicon sequencing ana­lysis were deposited in the DDBJ nucleotide sequence database under accession number DRA006974. The sequence reads of each OTU are available under accession numbers LC390430 to LC401807 and IADF01000001 to IADF01000317 for the 16S rRNA gene and *nosZ*, respectively.

## Results

### pH and temperature ranges and affinity for N_2_O reduction by G. aurantiaca

**Fig. 1.**
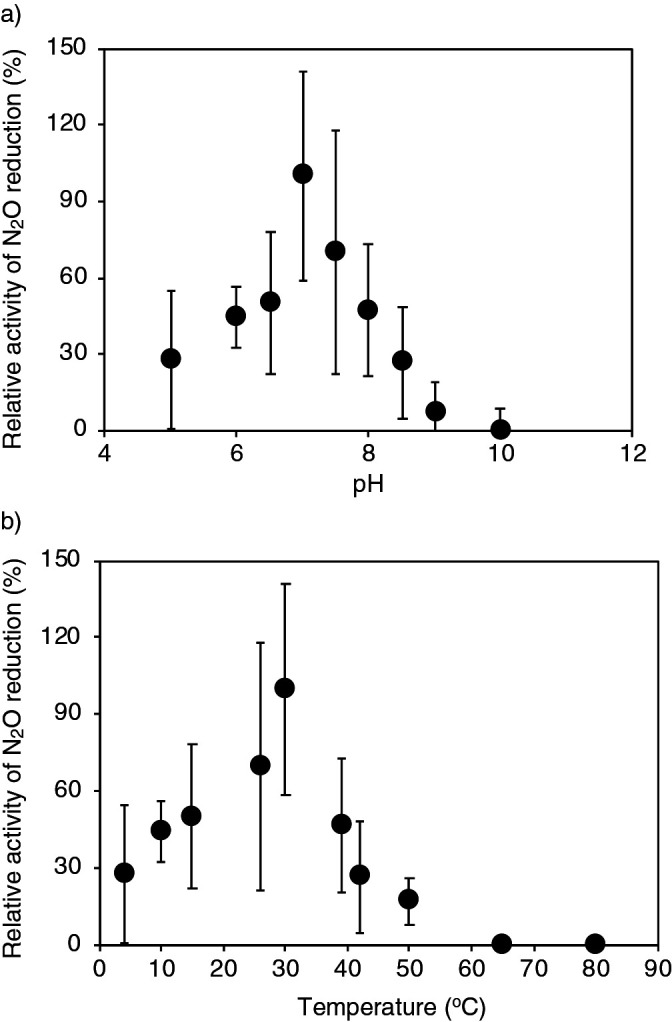
Effects of pH and temperature on N_2_O reduction by *Gemmatimonas aurantiaca* **a)** *G. aurantiaca* cells (a 3-mL sample in a 7.7-mL glass vial) were incubated at 30°C and a pH range of 5–10 with ^14-14^N_2_O (180‍ ‍nmol [N vial]^–1^). The highest activity, 0.0377‍ ‍nmol min^–1^ [mg protein]^–1^, was observed at pH 7. **b)** The incubation was repeated at pH 7 and 4–80°C. The highest activity, 0.0203‍ ‍nmol min^–1^ (mg protein)^–1^, was observed at 30°C. Error bars represent the range of standard deviations derived from three replicated vials.

**Fig. 2.**
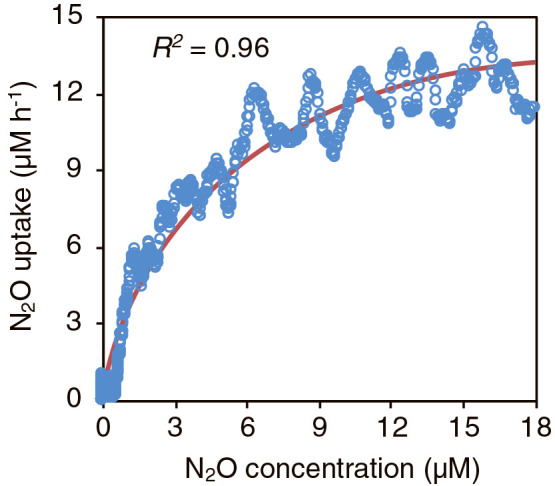
Affinity constant of *Gemmatimonas aurantiaca* for N_2_O reduction. *G. aurantiaca* cells were cultivated with the addition of 30‍ ‍μM N_2_O, and N_2_O consumption was monitored using a N_2_O microsensor. Circle symbols correspond to the data set obtained by N_2_O microsensor measurements, and the red line indicates a fitted Michaelis-Menten curve. The coefficient of determination (*R^2^*) was calculated at a range of 0.1 to 18‍ ‍μM N_2_O.

*G. aurantiaca* cells were incubated under different pH (pH 5 to 10) and temperature (4 to 80°C) conditions, and the activities of N_2_O consumption were examined. As shown in Fig. 1a and b, *G. aurantiaca* cells consumed N_2_O at pH 5–9 and 4–50°C, with the highest activity being observed at pH 7 and 30°C. The affinity of *G. aurantiaca* cells for N_2_O was examined by continuously measuring N_2_O concentrations using N_2_O-MR. The relationship between N_2_O reduction rates and N_2_O concentrations is shown in Fig. 2, and the apparent affinity constant for N_2_O (*K_s_*) was 4.4‍ ‍μM.

### N_2_O reduction activities and abundance of Gemmatimonadetes in soil

**Fig. 3.**
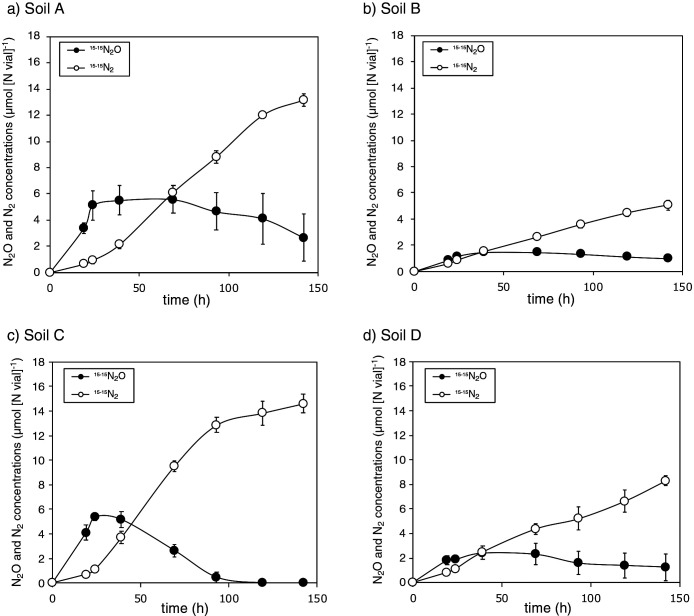
NO_3_^–^ reduction to N_2_O and N_2_ during the batch incubation of agricultural soil samples. Agricultural soils (Soils A to D) were incubated in closed 50-mL glass vials with the addition of ^15^NO_3_^–^ (71.4‍ ‍μmol [N vial]^–1^), and the production of ^15-15^N_2_O (filled circles) and ^15-15^N_2_ (open circles) was examined. Error bars represent the range of standard deviations derived from four replicated incubations.

Four agricultural soil samples collected in Nagaoka city, Niigata, Japan were incubated with the addition of ^15^NO_3_^–^. All soil samples reduced ^15^NO_3_^–^ and produced ^15-15^N_2_O and ^15-15^N_2_ (Fig. 3). N_2_O production was more prominent than N_2_ production during the early phase of the incubation (up to 24 to 69 h), and N_2_O concentrations then stabilized or decreased. In contrast, ^15-15^N_2_ concentrations continuously increased during the incubation, except for Soil C, which showed a decreased after an incubation for 93 h. ^15-15^N_2_O reduction rates were calculated as a slope of ^15-15^N_2_ concentrations during 0 to 93 h of the incubation, and were 16.6, 5.0, 24.3, and 9.5‍ ‍nmol N (g dry soil)^–1^ h^–1^ for Soil A, B, C, and D, respectively. These rates were similar or higher than those of Chinese paddy soils (2.37 to 8.31‍ ‍nmol N g^–1^ h^–1^) (Shan *et al.*, 2016).

**Fig. 4.**
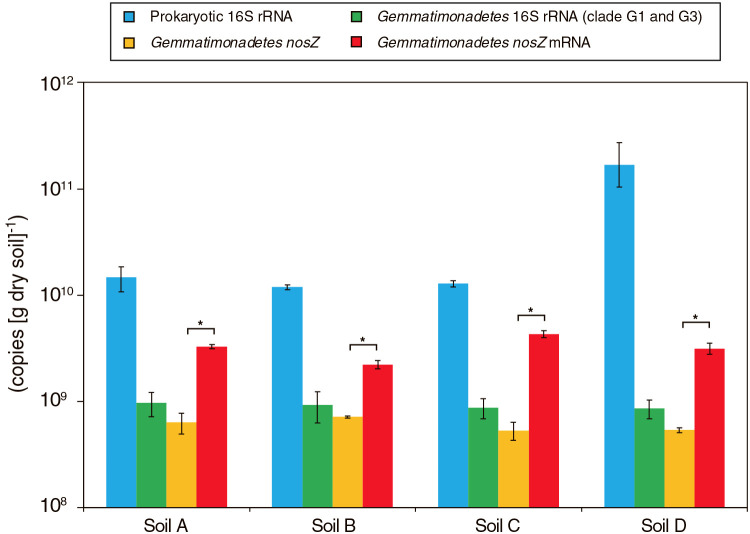
Abundance of 16S rRNA and *nosZ* in agricultural soil samples assessed by quantitative PCR (qPCR). Error bars represent the range of standard deviations derived from quadruplicate qPCR assays. No amplicon was obtained from the negative controls (*i.e.*, distilled water and the RNA template that was not reverse transcribed). A significant difference (Student’s *t-*test, 99% confidence interval) was observed between the copy numbers of *Gemmatimonadetes nosZ* DNA and mRNA, as shown with asterisks.

**Fig. 5.**
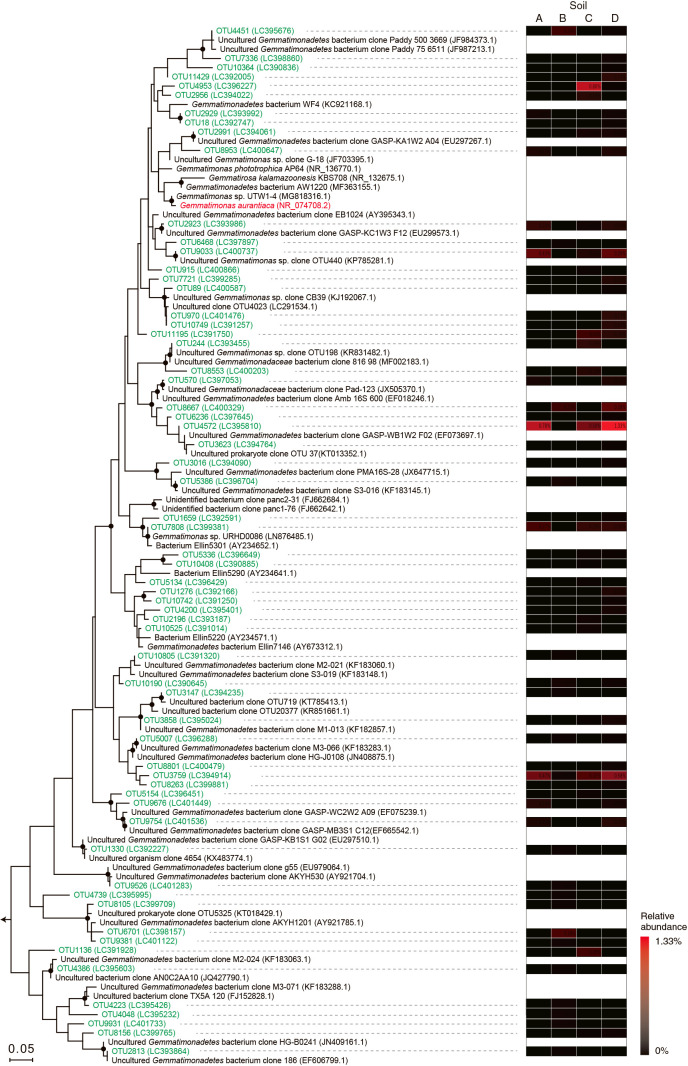
Phylogeny and abundance of *Gemmatimonadetes* 16S rRNA sequences detected in agricultural soil samples. 16S rRNA gene reads obtained by amplicon sequencing were clustered into species-level operational taxonomic units (OTUs) with ≥97% sequence identity, and a phylogenetic tree was constructed using the maximum likelihood method with the Jones-Taylor-Thornton model and the 16S rRNA of *Escherichia coli* (accession number BA000007.2) as an outgroup. Branching points that support a probability >80% in bootstrap ana­lyses (based on 500 replicates) are shown as filled circles. The scale bar represents 5% sequence divergence.

The abundance and diversity of the *Gemmatimonadetes* 16S rRNA gene and *nosZ* (DNA and mRNA) were examined using the above 4 soil samples collected after an incubation for 69 h. The copy numbers of the prokaryotic and *Gemmatimonadetes* (clades G1 and G3) 16S rRNA genes were 1.19–16.7×10^10^ and 8.62–9.65×10^8^ copies (g dry soil)^–1^, respectively (Fig. 4). The new oligonucleotide primers *nosZ*-123-145-F and *nosZ*-481-499-R were designed to assess the copy number of *Gemmatimonadetes nosZ*, and the sequence coverage of the designed primers is shown in Fig. S1. The copy numbers of *Gemmatimonadetes nosZ* DNA and mRNA (*i.e.*, cDNA) were 5.35–7.15×10^8^ and 2.23–4.31×10^9^ copies (g dry soil)^–1^, respectively (Fig. 4). PCR amplicons of the prokaryotic 16S rRNA gene and *Gemmatimonadetes nosZ* mRNA were subjected to an amplicon sequencing ana­lysis. Overall, 6,572 to 12,252 sequence reads of the 16S rRNA gene were obtained from each soil sample and then clustered based on ≥97% sequence identity into 2,982 to 4,021 OTUs (Table S2a). *Gemmatimonadetes* 16S rRNA reads accounted for 4.7 to 8.9% of the total reads (Fig. S2), and the phylogeny of *Gemmatimonadetes* 16S rRNA reads is shown in Fig. 5. The *Gemmatimonadetes* 16S rRNA reads affiliated with OTU4572 and OTU3759 were abundant in the soils tested, whereas no OTU shared ≥97% sequence similarity with the *G. aurantiaca* 16S rRNA gene sequence.

**Fig. 6.**
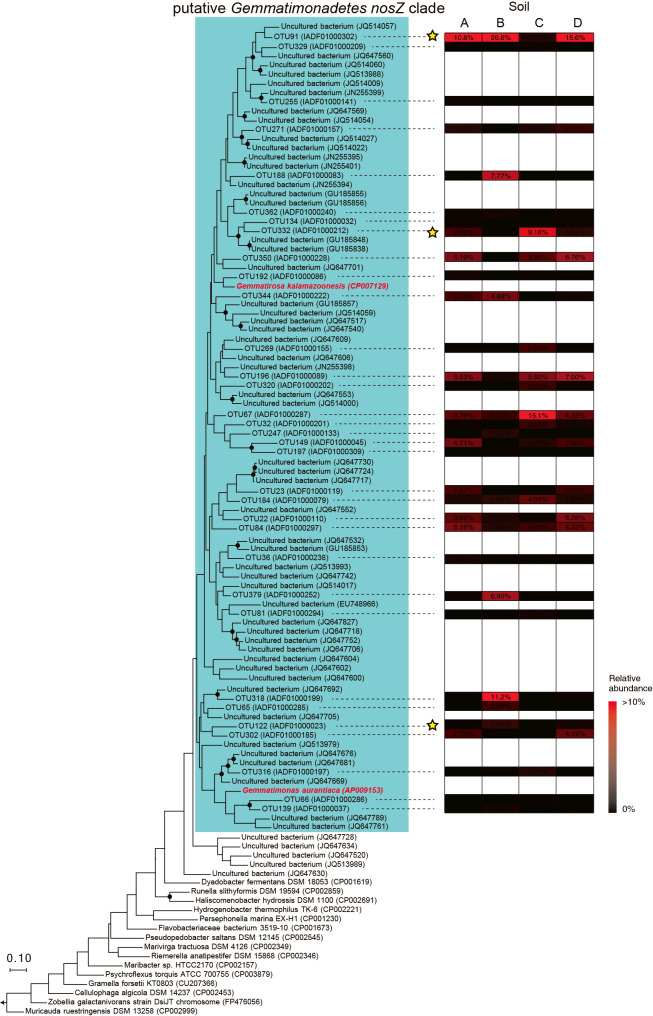
Phylogeny and abundance of 44 most abundant operational taxonomic units (OTUs) of *Gemmatimonadetes nosZ* mRNA. *nosZ* reads were clustered into species-level OTUs with ≥80% sequence identity (Palmer *et al.*, 2009), and a phylogenetic tree was constructed using the maximum likelihood method with the Jones-Taylor-Thornton model and *Robiginitalea biformata nosZ* (accession number; CP001712) as an outgroup. Branching points that support a probability >80% in bootstrap ana­lyses (based on 500 replicates) are shown as filled circles. The phylogenetic positions of *Gemmatimonadetes* *aurantiaca* and *Gemmatirosa kalamazoonesis nosZ* in the *Gemmatimonadetes nosZ* clade tentatively proposed in the present study are shown in red. The scale bar represents 10% sequence divergence. The OTUs highlighted with a star symbol (*i.e.*, OTU91, OTU332, and OTU122) are the major OTUs (>1% relative abundance in a soil sample) showing a strong correlation with soil N_2_O reduction rates (Table 1).

Regarding *Gemmatimonadetes nosZ* mRNA, 2,711 to 6,328 sequence reads were obtained (Table S2b). *Gemmatimonadetes nosZ* reads were screened from the total reads by a phylogenetic ana­lysis, and more than 72% of the total reads were affiliated with the *Gemmatimonadetes nosZ* clades; *i.e.*, 74, 86, 72, and 76% of *nosZ* reads obtained from Soil A, B, C, and D, respectively, were affiliated with a putative *Gemmatimonadetes nosZ* clade. *Gemmatimonadetes nosZ* reads were clustered (≥80% sequence identity) (Palmer *et al.*, 2009) into 90 *Gemmatimonadetes nosZ* OTUs. The phylogeny of the 44 major OTUs is shown in Fig. 6, and no OTU shared ≥80% sequence similarity with *G. aurantiaca nosZ*.

The relationships between N_2_O reduction rates and physicochemical parameters (Table S1) or the abundance of the *Gemmatimonadetes* 16S rRNA gene (clades G1 and G3) and *nosZ* were examined using a linear regression ana­lysis. As shown in Table 1, the abundance of the *nosZ* mRNA of *Gemmatimonadetes* bacteria, OTU91, OTU332, and OTU122 strongly correlated (*R^2^*; >0.84) with the N_2_O reduction rates of the soil samples tested.

## Discussion

The relative abundance of the *Gemmatimonadetes* 16S rRNA gene in the soils examined in the present study ranged between 0.5 and 7.8% of the prokaryotic 16S rRNA copy number and between 4.7 and 8.9% of the total 16S rRNA gene amplicon reads, indicating that *Gemmatimonadetes* bacteria were the dominant soil bacteria. The abundance of *Gemmatimonadetes* 16S rRNA gene copy numbers to prokaryotic 16S rRNA gene copy numbers assessed by qPCR was generally lower than the relative abundance estimated by the amplicon sequencing ana­lysis. The G1G3-673F primer used for qPCR of the *Gemmatimonadetes* 16S rRNA gene targeted the 16S rRNA gene sequences of *Gemmatimonadetes* clades G1 and G3, but not G2 (DeBruyn *et al.*, 2011), which may have resulted in an underestimation of the copy numbers of the *Gemmatimonadetes* 16S rRNA gene in the soils examined. On the other hand, although the *nosZ*-123-145-F and *nosZ*-481-499-R primers covered >92% of the *Gemmatimonadetes nosZ* sequences, the amplicon sequencing ana­lysis of *Gemmatimonadetes nosZ* mRNA revealed that between 14 and 28% of the total *nosZ* reads were not assigned to the putative *Gemmatimonadetes nosZ* clade. This result suggests that the copy numbers of *Gemmatimonadetes nosZ* mRNA (and potentially *nosZ* DNA) were overestimated by 28%. Therefore, caution is needed when comparing the copy numbers of the *Gemmatimonadetes* 16S rRNA gene and *nosZ*.

Previous DNA-based PCR and sequencing ana­lyses of *nosZ* clade II revealed metabolically inactive populations (*i.e.*, dead and dormant cells) in soil. *G. aurantiaca* and other N_2_O reducers transcribed *nosZ* mRNA when they reduced N_2_O (Henderson *et al.*, 2010; Mania *et al.*, 2016; Park *et al.*, 2017), and, thus, an mRNA-based ana­lysis of *Gemmatimonadetes nosZ* was herein performed to identify N_2_O-reducing *Gemmatimonadetes* bacterial populations. *Gemmatimonadetes nosZ* mRNA was successfully detected in N_2_O-reducing soils (Fig. 4 and 6), and correlations were observed between N_2_O reduction rates and the abundance of *Gemmatimonadetes nosZ* mRNA (*R^2^*=0.91). On the other hand, a weak correlation was noted between N_2_O reduction rates and the abundance of *Gemmatimonadetes nosZ* DNA (*R^2^*=0.36), which may have been due to the detection of a metabolically inactive *Gemmatimonadetes* population. Additionally, the abundance of the *Gemmatimonadetes* 16S rRNA gene (clades G1 and G3) did not show a correlation (*R^2^*=0.02). *nosZ* has been found in particular *Gemmatimonadetes* genomes and is not commonly conserved among this bacterial phylum, which may have contributed to the lack of a correlation between N_2_O reduction rates and the abundance of the *Gemmatimonadetes* 16S rRNA gene. The correlations observed between the N_2_O reduction rates of the soil samples tested and the abundance of the *nosZ* mRNA of *Gemmatimonadetes* bacteria, OTU91, OUT332, and OTU122 (Table 1) indicated the involvement of *Gemmatimonadetes* bacteria in N_2_O reduction in these soil samples. This is the first study to show a correlation between N_2_O reduction rates and the abundance of *Gemmatimonadetes nosZ* mRNA in soil. These correlations were examined using 4 agricultural soils in the present study, and, thus, more detailed studies are warranted using larger numbers and various types of soil samples. Furthermore, the soil samples examined in the present study contained phylogenetically diverse microorganisms other than *Gemmatimonadetes* bacteria (Fig. 2), and their contribution to N_2_O reduction and other nitrogen transformation reactions in soils currently remains unclear. A metatranscriptomic ana­lysis other than target-specific qPCR is a powerful tool for investigating microbial nitrogen transformation reactions in soil ecosystems (Masuda *et al.*, 2017).

To gain further insights into N_2_O reduction by *Gemmatimonadetes* bacteria in soil, the effects of pH and temperature as well as affinity for N_2_O reduction were examined using *G. aurantiaca*. The influence of pH and temperature on the N_2_O reduction activities of *Gemmatimonadetes* bacteria have not yet been examined; nevertheless, this physiological information is key for understanding their involvement in N_2_O reduction in soils. pH and temperature conditions markedly affected the N_2_O reduction activities of *G. aurantiaca* in the present study; relative activity decreased by 50±28% at pH 6.5 (Fig. 1). *G. aurantiaca* reduced N_2_O to N_2_ at pH 5–9 and 4–50°C, and agricultural soils showed these pH and temperature ranges; *i.e.*, pH 5.4–8.1 (Holtan-Hartwig *et al.*, 2000; Domeignoz-Horta *et al.*, 2015) and 3.6–25.8°C (Takata *et al.*, 2011). The *Ks* value for N_2_O (*i.e.*, 4.4‍ ‍μM) was in the range of those previously reported in cultures of denitrifiers and bacteria catalyzing dissimilatory nitrite reduction to ammonium (0.324–100‍ ‍μM) (Betlach and Tiedje, 1981; Conrad, 1996; Yoon *et al.*, 2016; Park *et al.*, 2017; Suenaga *et al.*, 2018) and using bulk agricultural soils (0.1–5.8‍ ‍μM) (Holtan-Hartwig *et al.*, 2000). N_2_O concentrations in agricultural soils were generally less than 1‍ ‍μM, but increased up to 400‍ ‍μM (Schreiber *et al.*, 2012 and references therein) with nitrogenous fertilizer treatment, which was higher than the *K*_s_ value of *G. aurantiaca*. Collectively, these findings indicate that soil management practices, including pH and temperature control and nitrogenous fertilizer treatment, have a significant impact on N_2_O reduction by *Gemmatimonadetes* bacteria (and likely N_2_O emissions from soil). Although the aforementioned physiological characteristics of *G. aurantiaca* support our hypothesis that *Gemmatimonadetes* bacteria participated in N_2_O reduction in the soils examined, it is important to note that the *G. aurantiaca* 16S rRNA gene and *nosZ* mRNA reads were not detected in the soil samples examined (Fig. 5 and 6); therefore, the physiological characteristics of soil-inhabiting *Gemmatimonadetes* bacteria need to be investigated in future studies. The 16S rRNA gene reads affiliated with OTU4572 and OTU3759 were commonly and abundantly found in the soils examined, and also from various types of European, American, and Asian soils as assessed by a blastn search against the nr database (Table S3a and b). Further studies are warranted to examine the physiological characteristics of the *Gemmatimonadetes* bacteria affiliated with OTU4572 and OTU3759.

## Citation

Oshiki, M., Toyama, Y., Suenaga, T., Terada, A., Kasahara, Y., Yamaguchi, T., and Araki, N. (2022) N_2_O Reduction by *Gemmatimonas aurantiaca* and Potential Involvement of *Gemmatimonadetes* Bacteria in N_2_O Reduction in Agricultural Soils. *Microbes Environ ***37**: ME21090.

https://doi.org/10.1264/jsme2.ME21090

## Supplementary Material

Supplementary Material

## Figures and Tables

**Table 1.** Relationships between N_2_O reduction rates and physicochemical parameters or the abundance of the *Gemmatimonadetes* 16S rRNA gene and *nosZ* in soil samples. The table shows *R^2^* values between the N_2_O reduction rates of the soil samples examined and (upper) physicochemical parameters or (bottom) the abundance of the *Gemmatimonadetes* 16S rRNA gene and *nosZ*. Regarding *Gemmatimonadetes nosZ* mRNA OTUs, the major OTUs (>1% of relative abundance in a soil sample, Fig. 6) with *R^2^* values >0.8 are shown.

pH	Water content	TC	TN	P	NO_3_^–^	NO_2_^–^	NH_4_^+^
0.09	0.57	0.58	0.62	0.03	0.42	0.41	0.41

Prokaryotic 16S rRNA gene	*Gemmatimonadetes*					
	16S rRNA gene	*nosZ* DNA	*nosZ* mRNA	OTU91 *nosZ* mRNA	OTU332 *nosZ* mRNA	OTU122 *nosZ* mRNA
0.11	0.02	0.36	0.91	0.97	0.84	0.92
