# Supplementary material for: N2O Reduction by Gemmatimonas aurantiaca and Potential Involvement of Gemmatimonadetes Bacteria in N2O Reduction in Agricultural Soils
Source: Microbes Environ. 2022 Apr 12;37(2):ME21090. doi: 10.1264/jsme2.ME21090 (PMC9530729; doi:10.1264/jsme2.ME21090)
Supplement: Supplementary file 1 — Supplementary Material [file 37_21090_s1.pdf]

Supplementary material

N_2O reduction by *Gemmatimonas aurantiaca* and potential involvement of *Gemmatimonadetes* bacteria in N_2O reduction in agricultural soils

Mamoru Oshiki, Yuka Toyama, Toshikazu Suenaga, Akihiko Terada, Yasuhiro Kasahara, Takashi Yamaguchi, and Nobuo Araki

The supplementary material contains 2 supplementary figures and 2 supplementary tables.

(a) *nosZ*-126-145-F

		1	2	3	4	5	6	7	8	9	10	11	12	13	14	15	16	17	18	19	20
primer (5' -3')		A	A	C	A	A	G	A	T	C	A	C	S	A	A	G	G	A	Y	C	G
nosZ alignment	A	97%	100%	0%	100%	100%	0%	100%	1%	0%	100%	0%	3%	100%	100%	8%	0%	100%	0%	5%	0%
	T	0%	0%	0%	0%	0%	0%	0%	97%	0%	0%	0%	0%	0%	0%	0%	0%	0%	38%	0%	0%
	G	3%	0%	0%	0%	0%	100%	0%	0%	0%	0%	0%	66%	0%	0%	92%	100%	0%	0%	0%	100%
	C	0%	0%	100%	0%	0%	0%	0%	1%	100%	0%	100%	31%	0%	0%	0%	0%	0%	62%	95%	0%
	Coverage	97%	100%	100%	100%	100%	100%	100%	97%	100%	100%	100%	97%	100%	100%	92%	100%	100%	100%	95%	100%

(b) *nosZ*-481-499-R

		1	2	3	4	5	6	7	8	9	10	11	12	13	14	15	16	17
primer (5' -3')		A	T	R	T	C	C	C	A	R	T	C	C	T	G	Y	T	C
nosZ alignment	A	100%	0%	12%	0%	0%	0%	0%	100%	23%	100%	0%	1%	0%	0%	13%	0%	0%
	T	0%	100%	0%	100%	0%	0%	0%	0%	0%	0%	0%	0%	100%	0%	0%	100%	0%
	G	0%	0%	88%	0%	0%	0%	0%	0%	77%	0%	100%	99%	0%	100%	87%	0%	0%
	C	0%	0%	0%	0%	100%	100%	100%	0%	0%	0%	0%	0%	0%	0%	0%	0%	100%
	Coverage	100%	100%	100%	100%	100%	100%	100%	100%	100%	100%	100%	99%	100%	100%	100%	100%	100%

100% >95% >90%

Fig. S1 (Oshiki et al.)

Figure S1 Primer-temperate mismatches between the nosZ-126-145F (a) and nosZ-481-499R (b) primers and *nosZ* sequences affiliated with the *Gemmatimonadetes nosZ* clade (See Fig. 6). Alignment positions of primers nosZ-126-145F and nosZ-481-499R in the *Gemmatimonas aurantiaca nosZ* sequence (accession number AP009153.1) were 1417 to 1436 and 1771 to 1787, respectively. The 77 *Gemmatimonadetes nosZ* sequences were retrieved by blastn search using *Gemmatimonas aurantiaca nosZ* as a query sequence against the nr database of NCBI. The row "Coverage" indicates sequence coverage of the examined *Gemmatimonadetes nosZ* primer at the specific position, and the heatmap highlights the regions showing high sequence coverage, i.e., red for 100% coverage, magenta for >95% coverage, and pink for >90% coverage.

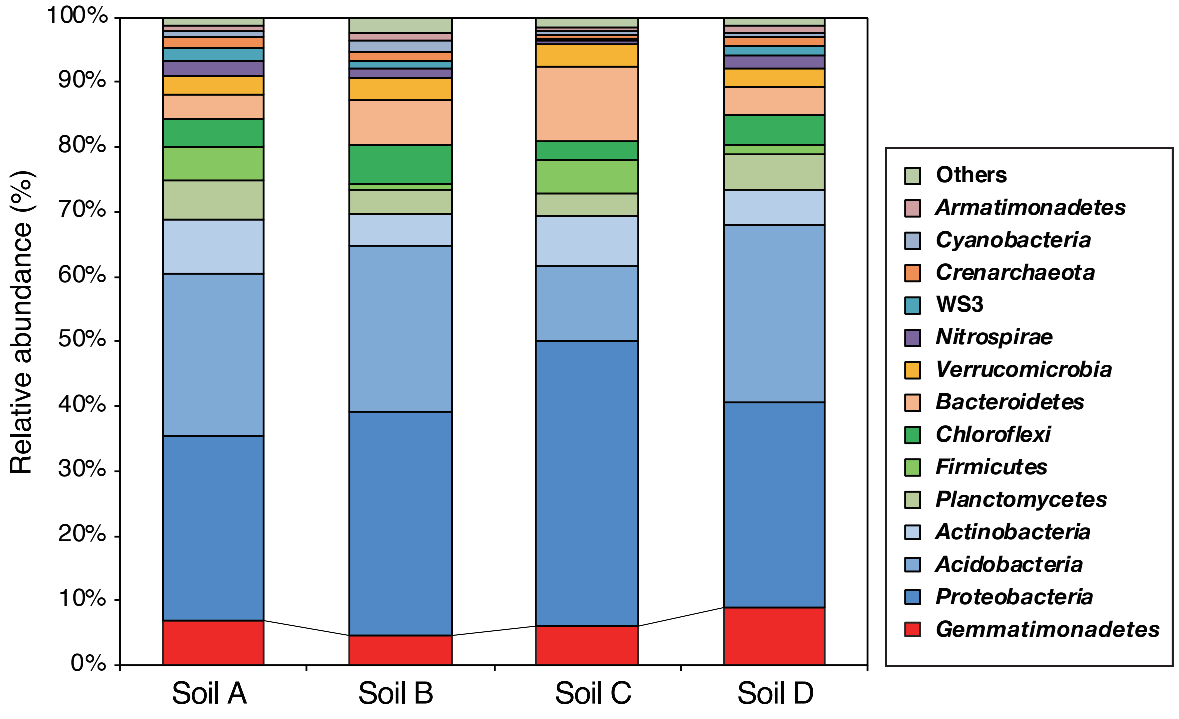

Fig. S2 (Oshiki et al.)

Figure S2 Taxonomic classification of prokaryotic communities in the studied agricultural soils. The reads that were not classified into any known prokaryotic group are labeled as ‘other.’.

Table S1. Description of the agricultural soil samples examined in the present study. TC; total carbon, TN; total nitrogen, P, NO_3^- and NO_2^-; 0.002N H_2SO_4-extractable phosphorus, nitrate and nitrite. NH_4^+; 0.5M sodium acetate-extractable ammonium.

	pH	Water contents (%)	TC (g kg^{-1})	TN (gN kg^{-1})	P (mg kg^{-1})	NO_3^- (mgN kg^{-1})	NO_2^- (mgN kg^{-1})	NH_4^+ (mgN kg^{-1})
Soil A	6.6	43	20.2	2.1	0.25	0.45	0.72	5.2
Soil B	7.5	24	35.3	3.3	0.41	0.12	0.65	4.8
Soil C	7.2	43	24.4	2.2	0.38	0.66	0.38	2.4
Soil D	6.9	41	39.2	3.9	0.31	0.66	1	8.2

Table S2 Community richness, diversity, and evenness indices of the studied agricultural soils. a) DNA-based amplicon sequencing analysis of prokaryotic 16S rRNA gene sequences amplified using the oligonucleotide primers 515F and 806r. b) mRNA-based amplicon sequencing analysis of *Gemmatimonadetes nosZ* amplified using the oligonucleotide primers nosZ-126-145-F and nosZ-481-499-R. OTU: operational taxonomic unit.

a) 16S rRNA gene

	Reads	OTU	Chao1	Shannon	Good's coverage
Soil A	6,572	2,982	10,642	10.4	66%
Soil B	9,796	3,927	13,281	10.6	71%
Soil C	12,252	4,021	11,633	10.2	77%
Soil D	10,762	3,800	12,299	10.3	75%

b) *nosZ* mRNA

	Reads	OTU	Chao1	Shannon	Good's coverage
Soil A	2,929	126	159	5.15	99%
Soil B	4,589	203	254	4.81	99%
Soil C	2,711	148	222	5.17	98%
Soil D	6,328	151	177	5.00	99%

Table S3. Geographic distribution of partial 16S rRNA gene sequences affiliated with the *Gemmatimonadetes* OTU4572 (a) and OTU3759 (b), respectively.

a) OTU4572

e-value	Identity	Accession	Isolation source	Country
6.00E-119	99%	EU051942.1	savanna soil (0-10 cm) under mesquite	USA: Texas, near Vernon
6.00E-119	99%	EU051935.1	savanna soil (0-10 cm) uander mesquite	USA: Texas, near Vernon
9.00E-111	97%	EU051939.1	savanna soil (0-10 cm) under mesquite	USA: Texas, near Vernon
3.00E-117	99%	FJ479377.1	undisturbed tall grass prairie, top 5	USA: Oklahoma, Kessler Farm Biological Station
2.00E-112	98%	FJ478758.1	undisturbed tall grass prairie, top 5	USA: Oklahoma, Kessler Farm Biological Station
3.00E-117	99%	EU134872.1	soil from an undisturbed mixed grass	USA: Oklahoma, Kessler farm
3.00E-117	99%	EU134860.1	soil from an undisturbed mixed grass	USA: Oklahoma, Kessler farm
3.00E-117	99%	EU134849.1	soil from an undisturbed mixed grass	USA: Oklahoma, Kessler farm
3.00E-117	99%	EU134846.1	soil from an undisturbed mixed grass	USA: Oklahoma, Kessler farm
3.00E-117	99%	EU134823.1	soil from an undisturbed mixed grass	USA: Oklahoma, Kessler farm
3.00E-117	99%	EU134821.1	soil from an undisturbed mixed grass	USA: Oklahoma, Kessler farm
9.00E-117	99%	EU134856.1	soil from an undisturbed mixed grass	USA: Oklahoma, Kessler farm
3.00E-116	98%	EU134869.1	soil from an undisturbed mixed grass	USA: Oklahoma, Kessler farm
1.00E-108	97%	EU134820.1	soil from an undisturbed mixed grass	USA: Oklahoma, Kessler farm
6.00E-119	99%	FJ621069.1	sweetgum plantation soil	USA: Oak Ridge, TN
8.00E-118	99%	FJ621221.1	sweetgum plantation soil	USA: Oak Ridge, TN
7.00E-106	97%	FJ621019.1	sweetgum plantation soil	USA: Oak Ridge, TN
1.00E-121	100%	EF516384.1	grassland soil	USA: northern California, Angelo Coast Range
1.00E-121	100%	EF516164.1	grassland soil	USA: northern California, Angelo Coast Range
1.00E-121	100%	EF515911.1	grassland soil	USA: northern California, Angelo Coast Range
6.00E-119	99%	EF516206.1	grassland soil	USA: northern California, Angelo Coast Range

7.00E-112	97%	EF516485.1	grassland soil	USA: northern California, Angelo Coast Range
5.00E-101	97%	GU200943.1	Nevada test site free air carbon	USA: Nevada
2.00E-113	98%	JN795872.1	soil	USA: Moab, Utah
8.00E-118	99%	EF663697.1	grassland at the GASP KBS-LTER sampling	USA: Michigan
3.00E-117	99%	EF663440.1	grassland at the GASP KBS-LTER sampling	USA: Michigan
1.00E-121	100%	EF664715.1	forest at the GASP KBS-LTER sampling	USA: Michigan
6.00E-119	99%	EF663094.1	cropland on GASP KBS-LTER sampling	USA: Michigan
6.00E-119	99%	EF662857.1	cropland on GASP KBS-LTER sampling	USA: Michigan
6.00E-119	99%	EF662856.1	cropland on GASP KBS-LTER sampling	USA: Michigan
6.00E-119	99%	EF662635.1	cropland on GASP KBS-LTER sampling	USA: Michigan
6.00E-119	99%	EF662450.1	cropland on GASP KBS-LTER sampling	USA: Michigan
2.00E-113	98%	EF662794.1	cropland on GASP KBS-LTER sampling	USA: Michigan
3.00E-117	99%	FJ615950.1	agricultural field sample one month	USA: Michigan
1.00E-121	100%	EU297495.1	burned native tallgrass priarie	USA: Kansas, GASP KPBS-LTER sampling site
6.00E-119	99%	KY942571.1	Acid Mine Drainage contaminated	USA: Iron Springs Mining District, CO
5.00E-120	99%	KU966768.1	soil	USA: Illinois
3.00E-117	99%	AY917517.1	volcanic deposit from 1790	USA: Hawaii
1.00E-121	100%	EF074272.1	pasture	USA: Georgia, GASP Watkinsville sampling site
6.00E-119	99%	EF075249.1	pasture	USA: Georgia, GASP Watkinsville sampling site
6.00E-119	99%	EF075090.1	pasture	USA: Georgia, GASP Watkinsville sampling site
6.00E-119	99%	EF074821.1	pasture	USA: Georgia, GASP Watkinsville sampling site
6.00E-119	99%	EF074506.1	pasture	USA: Georgia, GASP Watkinsville sampling site
6.00E-119	99%	EF074500.1	pasture	USA: Georgia, GASP Watkinsville sampling site

6.00E-119	99%	EF074239.1	pasture	USA: Georgia, GASP Watkinsville sampling site
3.00E-117	99%	EF074898.1	pasture	USA: Georgia, GASP Watkinsville sampling site
3.00E-117	99%	EF074057.1	pasture	USA: Georgia, GASP Watkinsville sampling site
3.00E-117	99%	EF073697.1	pasture	USA: Georgia, GASP Watkinsville sampling site
1.00E-121	100%	DQ450800.1	saturated alpine tundra wet meadow soil	USA: Colorado, Rocky Mountain Front Range, Niwot
5.00E-120	99%	DQ450801.1	saturated alpine tundra wet meadow soil	USA: Colorado, Rocky Mountain Front Range, Niwot
5.00E-120	99%	DQ450799.1	saturated alpine tundra wet meadow soil	USA: Colorado, Rocky Mountain Front Range, Niwot
2.00E-118	99%	DQ450798.1	saturated alpine tundra wet meadow soil	USA: Colorado, Rocky Mountain Front Range, Niwot
6.00E-119	99%	AY192278.1	Rocky Mountain alpine soil	USA: Colorado
6.00E-119	99%	FJ712883.1	Arabidopsis thaliana rhizosphere soil	USA: Center for Agricultural Research,
4.00E-109	100%	AY989238.1	soil	USA: Alaska
6.00E-119	99%	AY988875.1	soil	USA: Alaska
6.00E-119	99%	AY988685.1	soil	USA: Alaska
6.00E-119	99%	AY988629.1	soil	USA: Alaska
3.00E-117	99%	AY989225.1	soil	USA: Alaska
3.00E-117	99%	AY988740.1	soil	USA: Alaska
3.00E-117	99%	AY988725.1	soil	USA: Alaska
3.00E-117	99%	AY988719.1	soil	USA: Alaska
1.00E-115	99%	AY989156.1	soil	USA: Alaska
4.00E-115	99%	AY988806.1	soil	USA: Alaska
2.00E-113	99%	AY989589.1	soil	USA: Alaska
2.00E-113	99%	AY989391.1	soil	USA: Alaska
2.00E-113	99%	AY989370.1	soil	USA: Alaska
2.00E-113	99%	AY988758.1	soil	USA: Alaska
6.00E-113	99%	AY988759.1	soil	USA: Alaska

6.00E-113	99%	AY988709.1	soil	USA: Alaska
7.00E-112	99%	AY989409.1	soil	USA: Alaska
7.00E-112	99%	AY988655.1	soil	USA: Alaska
3.00E-111	99%	AY989294.1	soil	USA: Alaska
3.00E-111	99%	AY988890.1	soil	USA: Alaska
4.00E-109	99%	AY989155.1	soil	USA: Alaska
2.00E-107	99%	AY988936.1	soil	USA: Alaska
2.00E-106	99%	AY989325.1	soil	USA: Alaska
1.00E-103	99%	AY988965.1	soil	USA: Alaska
2.00E-100	99%	AY988843.1	soil	USA: Alaska
1.00E-108	97%	AY989083.1	soil	USA: Alaska
5.00E-120	99%	JF829170.1	permafrost near Kuparuk River and	USA: Alaska
5.00E-120	99%	JX859915.1	permafrost	USA: Alaska
6.00E-119	99%	KP910314.1	soil or post-volcanic pyroclastic	USA
6.00E-119	99%	KP906973.1	soil or post-volcanic pyroclastic	USA
5.00E-114	99%	KP923915.1	soil or post-volcanic pyroclastic	USA
1.00E-115	98%	KP937272.1	soil or post-volcanic pyroclastic	USA
7.00E-112	97%	KP904582.1	soil or post-volcanic pyroclastic	USA
9.00E-111	97%	KP935335.1	soil or post-volcanic pyroclastic	USA
9.00E-111	97%	KP929132.1	soil or post-volcanic pyroclastic	USA
9.00E-111	97%	KP927794.1	soil or post-volcanic pyroclastic	USA
1.00E-108	97%	KP934877.1	soil or post-volcanic pyroclastic	USA
1.00E-108	97%	KP921896.1	soil or post-volcanic pyroclastic	USA
6.00E-119	99%	MF015949.1	soil	USA
6.00E-119	99%	MF004972.1	soil	USA
3.00E-117	99%	HQ119139.1	loamy soils of Eucalyptus forest in La Jolla, CA	USA
3.00E-116	99%	HQ118726.1	loamy sand of Eucalyptus forest in La Jolla, CA	USA
5.00E-114	98%	HQ118781.1	loamy sand of Eucalyptus forest in La Jolla, CA	USA
7.00E-112	97%	HQ118761.1	loamy sand of Eucalyptus forest in La Jolla, CA	USA

6.00E-119	99%	EF073022.1	GASP Watkinsville sampling site, Georgia, USA	USA
3.00E-117	99%	EF073002.1	GASP Watkinsville sampling site, Georgia, USA	USA

b) OTU3759

e-value	Identity	Accession	Isolation source	Country
5.00E-120	99%	JX223173.1	subsurface aquifer sediment	USA: Rifle, Colorado
4.00E-109	97%	FJ479166.1	undisturbed tall grass prairie, top 5 cm	USA: Oklahoma, Kessler Farm Biological Station
1.00E-121	100%	EU134826.1	soil from an undisturbed mixed grass prairie preserve	USA: Oklahoma, Kessler farm
1.00E-121	100%	EU134800.1	soil from an undisturbed mixed grass prairie preserve	USA: Oklahoma, Kessler farm
5.00E-120	99%	EU134876.1	soil from an undisturbed mixed grass prairie preserve	USA: Oklahoma, Kessler farm
5.00E-120	99%	EU134868.1	soil from an undisturbed mixed grass prairie preserve	USA: Oklahoma, Kessler farm
5.00E-120	99%	EU134842.1	soil from an undisturbed mixed grass prairie preserve	USA: Oklahoma, Kessler farm
6.00E-119	99%	EU134832.1	soil from an undisturbed mixed grass prairie preserve	USA: Oklahoma, Kessler farm
6.00E-119	99%	EU134815.1	soil from an undisturbed mixed grass prairie preserve	USA: Oklahoma, Kessler farm
3.00E-116	98%	EU134880.1	soil from an undisturbed mixed grass prairie preserve	USA: Oklahoma, Kessler farm
1.00E-114	98%	EU134818.1	soil from an undisturbed mixed grass prairie preserve	USA: Oklahoma, Kessler farm
1.00E-114	98%	EU134810.1	soil from an undisturbed mixed grass prairie preserve	USA: Oklahoma, Kessler farm
2.00E-113	98%	EU134825.1	soil from an undisturbed mixed grass prairie preserve	USA: Oklahoma, Kessler farm
2.00E-112	98%	EU133059.1	soil from an undisturbed mixed grass prairie preserve	USA: Oklahoma, Kessler farm
7.00E-112	97%	EU134885.1	soil from an undisturbed mixed grass prairie preserve	USA: Oklahoma, Kessler farm
7.00E-112	97%	EU134884.1	soil from an undisturbed mixed grass prairie preserve	USA: Oklahoma, Kessler farm

3.00E-111	97%	EU134896.1	soil from an undisturbed mixed grass prairie preserve	USA: Oklahoma, Kessler farm
1.00E-109	97%	EU134841.1	soil from an undisturbed mixed grass prairie preserve	USA: Oklahoma, Kessler farm
5.00E-120	99%	EF516879.1	grassland soil	USA: northern California, Angelo Coast Range
5.00E-120	99%	EF516702.1	grassland soil	USA: northern California, Angelo Coast Range
1.00E-121	100%	JQ401127.1	soil	USA: near Moab, UT
5.00E-120	99%	JQ401206.1	soil	USA: near Moab, UT
5.00E-120	99%	JQ401153.1	soil	USA: near Moab, UT
5.00E-120	99%	JQ401151.1	soil	USA: near Moab, UT
5.00E-120	99%	JQ401150.1	soil	USA: near Moab, UT
5.00E-120	99%	JQ401149.1	soil	USA: near Moab, UT
5.00E-120	99%	JQ401129.1	soil	USA: near Moab, UT
5.00E-120	99%	JQ401126.1	soil	USA: near Moab, UT
6.00E-119	99%	JQ401154.1	soil	USA: near Moab, UT
6.00E-119	99%	JN795705.1	soil	USA: Moab, Utah
5.00E-120	99%	AY921859.1	farm soil adjacent to a silage storage bunker	USA: Minnesota
6.00E-119	99%	AY922128.1	farm soil adjacent to a silage storage bunker	USA: Minnesota
6.00E-119	99%	AY921922.1	farm soil adjacent to a silage storage bunker	USA: Minnesota
3.00E-117	99%	AY921860.1	farm soil adjacent to a silage storage bunker	USA: Minnesota
1.00E-109	97%	AY921751.1	farm soil adjacent to a silage storage bunker	USA: Minnesota
1.00E-109	97%	AY921749.1	farm soil adjacent to a silage storage bunker	USA: Minnesota
1.00E-121	100%	EF663955.1	grassland at the GASP KBS-LTER sampling site	USA: Michigan
6.00E-119	99%	EF664055.1	grassland at the GASP KBS-LTER sampling site	USA: Michigan

5.00E-120	99%	EF663342.1	cropland on GASP KBS-LTER sampling site	USA: Michigan
5.00E-120	99%	EF663106.1	cropland on GASP KBS-LTER sampling site	USA: Michigan
2.00E-113	98%	EF663198.1	cropland on GASP KBS-LTER sampling site	USA: Michigan
5.00E-120	99%	FJ615963.1	agricultural field sample one month after swine manure application	USA: Michigan
9.00E-111	97%	GQ500725.1	Charon's Cascade, sandy clastic sediments	USA: Mammoth Cave, Kentucky
6.00E-113	98%	EU528223.1	sediment	USA: Kentucky, Kentucky Lake
5.00E-120	99%	EU297024.1	cropland	USA: Kansas, GASP KPBS-LTER sampling site
5.00E-120	99%	EU296949.1	cropland	USA: Kansas, GASP KPBS-LTER sampling site
3.00E-116	98%	EU297325.1	cropland	USA: Kansas, GASP KPBS-LTER sampling site
6.00E-119	99%	EU297706.1	burned native tallgrass priarie	USA: Kansas, GASP KPBS-LTER sampling site
7.00E-112	97%	EU300225.1	2000 restored grassland	USA: Kansas, GASP KPBS-LTER sampling site
2.00E-113	98%	EU299631.1	1998 restored grassland	USA: Kansas, GASP KPBS-LTER sampling site
5.00E-120	99%	KY943207.1	Acid Mine Drainage contaminated sediments	USA: Iron Springs Mining District, CO
3.00E-117	99%	KY943595.1	Acid Mine Drainage contaminated sediments	USA: Iron Springs Mining District, CO
7.00E-112	97%	KY942889.1	Acid Mine Drainage contaminated sediments	USA: Iron Springs Mining District, CO
1.00E-109	97%	KY943683.1	Acid Mine Drainage contaminated sediments	USA: Iron Springs Mining District, CO
2.00E-113	98%	KU966301.1	soil	USA: Illinois
1.00E-109	97%	KU967165.1	soil	USA: Illinois

6.00E-119	99%	GQ263125.1	simulated low level waste site	USA: Idaho National Labs
6.00E-113	98%	GQ263009.1	simulated low level waste site	USA: Idaho National Labs
1.00E-121	100%	EF074299.1	pasture	USA: Georgia, GASP Watkinsville sampling site
5.00E-120	99%	EF075160.1	pasture	USA: Georgia, GASP Watkinsville sampling site
5.00E-120	99%	EF074615.1	pasture	USA: Georgia, GASP Watkinsville sampling site
2.00E-118	99%	EF073881.1	pasture	USA: Georgia, GASP Watkinsville sampling site
3.00E-111	98%	FJ712847.1	Arabidopsis thaliana rhizosphere soil from a fallow, experimental, agricultural site, not	USA: Center for Agricultural Research
4.00E-115	99%	HM988906.1	grassland soil	USA: California
4.00E-109	99%	EU280634.1	bulk soil from grassland	USA: California
6.00E-119	99%	EF612389.1	soil	USA: Arizona, Klondyke, Klondyke Mill Site
6.00E-119	99%	AY988853.1	soil	USA: Alaska
1.00E-115	99%	AY988608.1	soil	USA: Alaska
6.00E-113	99%	AY988869.1	soil	USA: Alaska
7.00E-112	99%	AY989632.1	soil	USA: Alaska
7.00E-112	99%	AY988969.1	soil	USA: Alaska
9.00E-111	99%	AY988952.1	soil	USA: Alaska
3.00E-110	99%	AY988881.1	soil	USA: Alaska
5.00E-108	97%	AY989592.1	soil	USA: Alaska
6.00E-113	98%	AY289482.1	soil, 0-7 cm subsurface	USA: Abbott's Pit, Virginia
1.00E-121	100%	KP908065.1	soil or post-volcanic pyroclastic surface	USA
6.00E-119	99%	KP911475.1	soil or post-volcanic pyroclastic surface	USA
3.00E-117	99%	KP912906.1	soil or post-volcanic pyroclastic surface	USA
2.00E-113	98%	KP908369.1	soil or post-volcanic pyroclastic surface	USA

2.00E-113	98%	KP907519.1	soil or post-volcanic pyroclastic surface	USA
2.00E-113	98%	KP906004.1	soil or post-volcanic pyroclastic surface	USA
2.00E-113	98%	KP905649.1	soil or post-volcanic pyroclastic surface	USA
9.00E-111	97%	KP908923.1	soil or post-volcanic pyroclastic surface	USA
9.00E-111	97%	KP908435.1	soil or post-volcanic pyroclastic surface	USA
1.00E-109	97%	KP913850.1	soil or post-volcanic pyroclastic surface	USA
1.00E-109	97%	KP913353.1	soil or post-volcanic pyroclastic surface	USA
1.00E-108	97%	KP935624.1	soil or post-volcanic pyroclastic surface	USA
5.00E-120	99%	MF004752.1	soil	USA
5.00E-120	99%	KX239116.1	soil	USA
3.00E-116	98%	MF014304.1	soil	USA
1.00E-114	98%	MF010331.1	soil	USA
2.00E-113	98%	MF004637.1	soil	USA
9.00E-111	97%	MF011029.1	soil	USA
9.00E-111	97%	MF005341.1	soil	USA
9.00E-111	97%	MF005308.1	soil	USA
2.00E-113	98%	KY254316.1	Sequenced from crayfish (Cambarus chasmodactylus) cuticle	USA
1.00E-109	97%	KY251654.1	Sequenced from crayfish (Cambarus chasmodactylus) cuticle	USA
5.00E-120	99%	HM186284.1	saturated zone of the Hanford Site 300	USA
6.00E-119	99%	HM186205.1	saturated zone of the Hanford Site 300	USA

2.00E-113	98%	HM186787.1	saturated zone of the Hanford Site 300	USA
